# Adolescents’ Well-Being and Democratic Parenting: Does Environmental Sensitivity Matter?

**DOI:** 10.3390/healthcare13060659

**Published:** 2025-03-17

**Authors:** Nicolò Maria Iannello, Nicla Cucinella, Alida Lo Coco, Sonia Ingoglia, Costanza Baviera, Cristiano Inguglia, Francesca Lionetti, Michael Pluess, Maria Grazia Lo Cricchio

**Affiliations:** 1 Department of Psychology and Health Sciences, Pegaso Telematic University, 80143 Naples, Italy; nicolomaria.iannello@unipegaso.it; 2 Department of Psychology, Educational Science and Human Movement, University of Palermo, 90128 Palermo, Italy; alida.lococo@unipa.it (A.L.C.); sonia.ingoglia@unipa.it (S.I.); cristiano.inguglia@unipa.it (C.I.); 3 Department of Research and Innovation in Humanities, University of Bari Aldo Moro, 70121 Bari, Italy; c.baviera@phd.uniba.it; 4 Department of Brain and Behavioral Sciences, University of Pavia, 27100 Pavia, Italy; francesca.lionetti@unipv.it; 5 Department of Psychological Sciences, School of Psychology, University of Surrey, Guildford GU2 7XH, UK; m.pluess@surrey.ac.uk; 6 Department of Humanistic, Scientific and Social Innovation, University of Basilicata, 85100 Potenza, Italy; mariagrazia.locricchio@unibas.it

**Keywords:** democratic parenting, environmental sensitivity, well-being, adolescence

## Abstract

**Background/Objectives:** This study examines the relations between democratic parenting and adolescents’ subjective well-being, focusing on the potential moderating role of youth environmental sensitivity. Three environmental sensitivity models (diathesis–stress, vantage sensitivity, differential susceptibility) were tested to unveil the heterogeneity of the effects of democratic parenting on adolescents’ subjective well-being according to the type of environmental influences (positive, negative, both positive and negative) youths are more susceptible to. **Methods:** A sample of 321 Italian adolescents (75% females; *M*_age_ = 16.83, *SD* = 1.35) completed online self-report measures assessing perceptions of democratic parenting, environmental sensitivity, and various dimensions of subjective well-being (positivity, vitality, anxiety, and general health). **Results:** Three path analysis models were run to evaluate the associations between democratic parenting, environmental sensitivity, and youths’ well-being. The results showed that democratic parenting was positively related to adolescents’ positivity, vitality, and general health and negatively to anxiety. Adolescents with higher environmental sensitivity reported reduced subjective well-being. A moderating effect of environmental sensitivity was detected such that highly sensitive adolescents had lower general health when democratic parenting was low. **Conclusions:** These findings support a diathesis–stress model, suggesting that environmental sensitivity might be a vulnerability factor in less supportive environments.

## 1. Introduction

Adolescence is a period of the lifecycle marked by rapid and critical bodily, cognitive, and social transformations that are impactful on youth’s psychological well-being [1,2,3]. In light of this, several lines of research have attempted to identify those factors that could sustain adolescents’ optimal mental and physical health in such a time of transition [4,5].

Among contextual variables, familial relationships based on openness, mutual listening, and respect are considered favorable resources from which adolescents might benefit for their healthy and successful growth [6,7]. Interestingly, it has been shown that a personal trait, such as sensitivity to environmental stimuli, might make adolescents more or less susceptible to both positive and negative experiences within their families [8,9].

However, it is understudied how the relationship between youths’ well-being and positive parenting might vary according to adolescents’ levels of environmental sensitivity. Indeed, previous studies in this domain mainly focused on negative parenting characteristics, such as parents’ rejection, and their undesirable outcomes, such as depression [10,11]. To shed light on the complex association between positive parenting, youths’ well-being, and environmental sensitivity, we conducted a study involving Italian adolescents. Therefore, we adopted a socio-ecological perspective on adolescents’ well-being, which suggests that it is the product of the interplay of personal and social factors [12].

### 1.1. Conceptualizing Well-Being

Well-being is a multidimensional concept that has been widely investigated both theoretically and empirically [13,14,15]. Theoretically speaking, two prominent approaches are generally used to define it: the hedonistic one, describing well-being as an internal state of happiness, and the eudaimonic one, based on the notion that well-being means more than just being happy rather, it is about fulfilling one’s potential and accomplishing personal growth [14,16]. Regarding youth well-being, a variety of domains and subdomains have been proposed to investigate this construct, including subjective and social features [4].

In the current study, the focus is on subjective components of adolescents’ well-being, that is, on “self-representations of intrapersonal affective or emotional states reflecting a sense of subjective well-being or distress” [17] (p. 5152). In detail, well-being has been investigated across four dimensions, namely positivity, vitality, anxiety, and general health. Positivity refers to people’s general tendency to view one’s life in a benign way [18]; vitality refers to experiencing aliveness and energy [19]; anxiety refers to excessive and uncontrollable worry and apprehension [20]; general health to optimal/dysfunctional bodily and mental conditions that might allow/impair individuals to fully engage in their activities [4,21,22]. We chose these specific elements of well-being since adolescents face several bodily (e.g., morphological transformations), personal (e.g., identity formation), and social changes (e.g., forming and managing new friendships and relationships) that might negatively impact their approach to and visions of life, enhance their levels of anxiety, and compromise their general health. We explored these dimensions of adolescents’ well-being by considering the impact that democratic parenting practices (contextual factor), environmental sensitivity (individual factor), and their interplay might have on them.

### 1.2. Democratic Parenting and Adolescents’ Well-Being

Relationships with parents have been largely shown to contribute to children’s positive and negative adjustment and development [23], according to the type of behaviors adopted by mothers and fathers in the care of their offspring [24]. Among the prototypical parenting styles, which have been identified by combining the dimensions of demandingness and responsiveness—that is, authoritarian (high demandingness, low responsiveness), indulgent (high responsiveness, low demandingness), and neglectful (low demandingness, low responsiveness)—the authoritative one seems to foster the most favorable developmental outcomes, as it balances demandingness, responsiveness, and independence [24,25,26,27].

As such, the authoritative parenting style might be considered a promising context for the promotion of democratic parent-adolescent relationships [28]. Indeed, in such a familial climate, youth might engage in discussions with their parents and voice their opinions. At the same time, parents might be more prone to meet their children’s needs and respect their points of view and autonomy [28,29,30].

There is evidence that democratic parenting positively impacts adolescents’ academic, emotional, and social well-being by encouraging youth disclosure [6]. Additionally, it has been reported that adolescents with congenital heart disease from democratic households exhibited the most favorable psychological and health behavior outcomes compared to those from psychologically controlling families [31]. One plausible explanation for the beneficial influences of democratic parenting on youth well-being is that democratic parents might make adolescents feel that they are supported and valued and make them experience a sense of connectedness to the familial milieu [6,29].

However, it is still understudied how a parenting style emphasizing communication, symmetry, and reciprocity might influence adolescents’ approach to life and their sense of aliveness and energy. In general, the literature has evidenced that a comfortable family context seems to play a part in the promotion of adolescents’ self-esteem, positive self-evaluations, and adjustment (for review, [32]), as well as of youths’ positive psychological capital [33]. Additionally, there is still a dearth of knowledge on the role of the interplay between democratic parenting and youth environmental sensitivity on adolescents’ well-being. To fill these gaps, the current study aimed at expanding knowledge on parent–child relationships by expressly focusing on the influences of democratic parenting on children’s well-being in terms of positivity, vitality, anxiety, and general health and by contemplating the moderator role of environmental sensitivity in such linkages.

### 1.3. Parenting Practices and Adolescents’ Well-Being: The Role of Environmental Sensitivity

Environmental sensitivity might be defined as the individual variation in perceiving, processing, and responding to experiential and contextual influences [34,35]. The construct has been largely investigated and shown to be made of three factors: *Aesthetic Sensitivity* (AES), referring to the response to aesthetic stimuli (e.g., being moved by good music); *Low Sensory Threshold* (LST), referring to unpleasant sensory arousal to external stimuli (e.g., being made uncomfortable by bright lights); and *Ease of Excitation* (EOE), concerning being overwhelmed by internal and external demands (e.g., having too much to do [36,37]).

One central notion of the frameworks describing environmental sensitivity is that individuals differ in their levels of sensitivity to the environment, meaning that some are more, and some are less susceptible to both positive and negative contextual influences [34]. Three different perspectives have proposed “different ideas about the type of environmental influences more environmentally sensitive individuals respond more strongly to” [38] (p. 674). The diathesis–stress (or dual-risk) model suggests that individuals who show higher environmental sensitivity suffer more when exposed to adverse environmental influences (so their susceptibility can be described as “for worse” [39,40]). Differently, the vantage sensitivity model highlights that individuals with higher sensitivity benefit more from positive, supportive environments (so, in this case, higher susceptibility is “for better” [41]). Finally, the differential susceptibility (“for better and for worse”) models, which include the Sensory Processing Sensitivity [42], suggest that environmental sensitivity makes individuals more disposed to suffer from adverse environments but, at the same time, to benefit more from supportive environments.

Whatever perspective is adopted, the literature has demonstrated that high sensitivity is related to anxiety and depression among undergraduate students [11]. Similarly, highly sensitive children have been found to report problems with their daily functioning (e.g., medically unexplained physical symptoms and internalizing problems) [43]. A plausible explanation for such associations is that hypersensitive individuals’ predisposition to deeply process information and to experience a more intricate inner, emotional life might lead them to negative feelings and ruminative thoughts, which set the stage for maladjustment and ill-being [43,44,45]. However, it might be posited that favorable or adverse consequences of environmental sensitivity on individuals’ well-being and adjustment might be intertwined with the quality of their contexts [44].

Relatedly, some lines of research have evidenced that highly sensitive children feel more socially competent when they experience a supportive family atmosphere [46]. In addition, it has been reported that low-quality parenting might expose more sensitive children to internalizing problems [44]. Thus, such findings recall a socio-ecological perspective on human development, positing that person–environment interactions might help explain individuals’ outcomes, such as their subjective well-being [12].

Although these findings are illuminating, the role of environmental sensitivity in moderating the effects of democratic parenting on adolescents’ subjective well-being is yet to be extensively explored. To further establish the consistency of the moderating effect of environmental sensitivity on this association, this study tested the three above-mentioned theoretical models. Therefore, we expected to unveil something more about the heterogeneity of the effects of democratic parenting on adolescents’ optimal development by accounting for the type of environmental influences (positive, negative, both positive and negative) youths are more susceptible to and more prone to respond to [38]. For example, when democratic parenting practices are low, adolescents who are more sensitive to adversities should be expected to report lower subjective well-being than those who are more sensitive to positive stimuli; on the contrary, when democratic parenting behaviors are high, adolescents who are more sensitive to positive stimuli should be expected to report higher levels of subjective well-being compared to those more sensitive to adversities. Finally, the subjective well-being of differentially susceptible adolescents should be expected to be more affected by both high and low levels of democratic parenting practices and behaviors.

### 1.4. Aims of the Study

The literature evidenced that changes and challenges occurring during adolescence at an individual and social level might have an impact on youth’s subjective well-being. Hence, a better understanding of factors that might foster well-being is fundamental to helping adolescents in their transition to adulthood. At the social level, democratic parenting may be a promotive factor for youths’ well-being; however, it is still understudied whether its beneficial influence may be conditioned by an individual factor, such as adolescents’ environmental sensitivity.

In light of this, our general goal was to explore the moderating role of adolescents’ environmental sensitivity in the association between their perception of democratic parenting and their well-being (in terms of positivity, vitality, general health, and anxiety). Based on previous considerations and in line with a socio-ecological perspective on adolescents’ well-being and with the environmental sensitivity framework, we propose the following hypotheses:

**H1.**  *Adolescents perceiving parents as more democratic in their parenting practices tend to exhibit higher levels of positivity, vitality, and general health, as well as lower levels of anxiety*.

**H2.**  *Adolescents who report higher levels of environmental sensitivity tend to report lower levels of positivity, vitality, and general health, as well as higher levels of anxiety*.

Regarding the moderating role of adolescents’ environmental sensitivity in the association between their perception of democratic parenting and their own well-being, this research evaluates three alternative hypotheses:

**H3a.**  *Highly* *sensitive* *adolescents* *are vulnerable to low democratic parenting only (the diathesis–stress model).*

**H3b.**  *Highly* *sensitive* *adolescents benefit more from high democratic parenting only (the vantage-sensitivity model).*

**H3c.**  *Highly* *sensitive* *adolescents are vulnerable to low democratic parenting and benefit more from high democratic parenting (the differential susceptibility model).*

**Figure 1:**
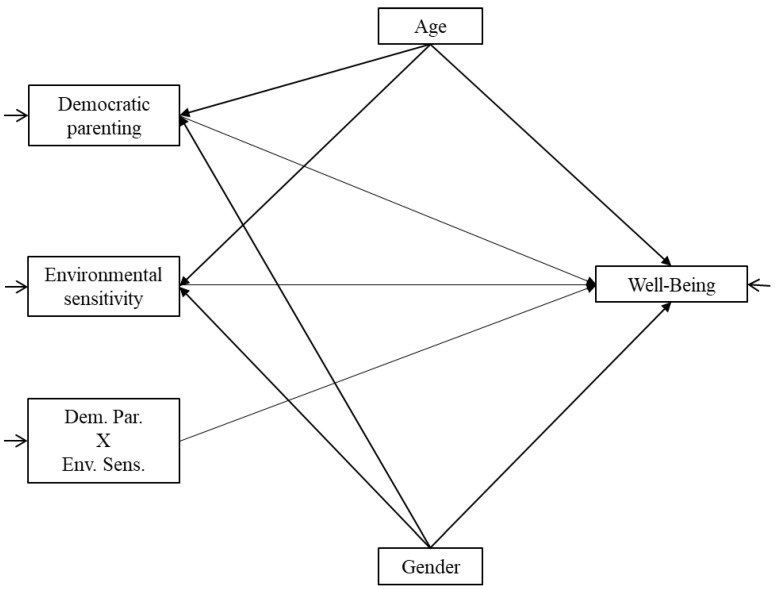
Hypothesized model for a generic well-being component.

The hypothesized model for a generic well-being component is depicted in Figure 1. To control the potential effects of gender and age, these variables were specified as covariates in the model.

## 2. Materials and Methods

### 2.1. Participants and Procedure

Participants were 321 Italian adolescents (75% females) aged between 14 and 19 years (*M* = 16.83; *SD* = 1.35), attending several high schools in Sicily (Southern Italy). Socio-demographic characteristics are reported in Table 1.

Participants were asked to answer the survey in an online format. We avoided missing data by introducing the mandatory response in the Google Module. Participants were ensured that their participation in the study was voluntary and that data would be treated confidentially and only used for scientific purposes. This was conducted through an information sheet, and participants were given the opportunity to ask questions about the background and the purpose of the study. Written informed consent was obtained from all adolescents, but firstly from their parents. Ethical permission to conduct the study was obtained from the University of Palermo (protocol code 119/2022). All procedures were carried out in accordance with the Declaration of Helsinki.

### 2.2. Measures

#### 2.2.1. Democratic Parenting

Adolescents were administered the Parental Democratic Functioning scale (PDF) [28]. It is a 6-item scale assessing teens’ perception of their parents’ use of democratic parenting practices (e.g., “My parents allow me to participate when decisions are being made in the family”). Items are rated on a 4-point Likert scale (from 1 = absolutely wrong for me to 4 = absolutely true for me). The total score is obtained as the sum of items scores. In the present study, the internal consistency was good (Cronbach’s α = 0.89).

#### 2.2.2. Environmental Sensitivity

Adolescents were administered the Highly Sensitivity Child Scale (HSCS) [36]; Italian adaptation by Nocentini et al. [47]. It is a 12-item scale articulated in three subscales: (a) Ease of Excitation, which refers to being easily overwhelmed by external and internal demands (5 items; e.g., “I find unpleasant to have a lot going on at once”); (b) Aesthetic Sensitivity, which captures the response and appreciation of aesthetic stimuli (4 items; e.g., “Some music can make me really happy”); and (c) Low Sensitivity Threshold, which reflects unpleasant sensory arousal to external stimuli (3 items; e.g., “I don’t like loud noises”). Items are rated on a 7-point Likert scale (from 1 = not at all to 7 = completely). In this study, we computed a total score of environmental sensitivity, which was obtained as the sum of item scores. In the present study, the internal consistency was adequate (for the total scale, Cronbach’s α = 0.74).

#### 2.2.3. Well-Being

Adolescents were administered four subscales of the Psychological General Well-Being Index (PGWB) [48], an Italian adaptation by Grossi et al. [49]. Specifically, we used the positive well-being subscale, which evaluates general feelings of cheerfulness, peacefulness, and satisfaction with one’s personal life (4 items, e.g., “In the last 4 weeks, to what extent have you felt happy, satisfied, or content with your personal life”?); the vitality subscale, which evaluates feelings of energy, rest, and activity (4 items, e.g., “In the last 4 weeks, I woke up feeling fresh and rested”); the general health subscale, which evaluates feelings of apprehension, worry, or fear for one’s own health (3 items, e.g., “In the last 4 weeks, have you experienced apprehension, worry, or fear about your health”?); and the anxiety subscale which evaluates feelings of tension, worry or pressure (4 items, e.g., “ In the last 4 weeks, have you been anxious, worried, or angry”?). Items were rated on a 6-point Likert scale (from 1 to 6). The subscale score is obtained as the sum of item scores for each subscale. In the present study, the internal consistency was acceptable for general health (α = 0.60) and adequate for other subscales: positive well-being, α = 0.82; vitality, α = 0.78; anxiety, α = 0.85.

### 2.3. Data Analysis

To test whether democratic parenting interacts with adolescent’s environmental sensitivity in predicting their well-being, three path analysis models were specified (one for each well-being component), with a predictor (democratic parenting), a moderator (environmental sensitivity), an interaction term (democratic parenting x environmental sensitivity), and an outcome (well-being component). Both the predictor and the moderator were standardized before computing the interaction term. We statistically controlled for gender and age effects on outcomes, predictors, and moderators. The Maximum Likelihood (ML) estimation method was used. The overall fit of each model was tested using several goodness-of-fit statistics and an evaluation of the appropriateness of the model parameters. The χ^2^ statistic assessed the implied covariance matrix compared with a good-fitting model indicated by a non-significant result. Given the potential limitation of the χ^2^ test (it should be non-significant with *p* > 0.05), due to its tendency to reject the null hypothesis with large sample sizes and complex models, we relied on well-known goodness-of-fit indices and their related cut-offs to evaluate model fit [50]: CFI ≥ 0.90 for acceptable and ≥0.95 for good fit, RMSEA ≤ 0.08 for acceptable and ≤0.05 for good fit. We probed interactions using simple slope analyses: simple slopes were tested at high and low levels of environmental sensitivity (±1 SD) consistent with recommended practices [51].

Two additional tests were carried out to assess whether interaction effects were consistent with differential-susceptibility, diathesis–stress, or vantage-sensitivity theory [52]. First, regions of significance (RoS) were examined to check whether well-being differences between teens high and low on environmental sensitivity were present for low democratic parenting only (diathesis–stress), high democratic parenting only (vantage-sensitivity), or for both high and low democratic parenting (differential susceptibility). This is referred to as the RoS on X test by Roisman et al. [53], who recommend that such differences should be observable within common values of the predictor/X variable of interest (i.e., ±2 SDs), in this case, democratic parenting. Second, because the RoS on the X test is sensitive to sample size, the proportion of interaction (PoI) was calculated to assess the proportion of the total interaction that is represented on the right side of the crossover point for the interaction. In differential susceptibility theory, this represents the area for which the effect of the predictor on the outcome is ‘for better’. In a prototypical differential susceptibility account, this value will be 50%. In a prototypical diathesis–stress account, this value will be 0%. Analyses were run with Mplus 7 [54].

## 3. Results

### 3.1. Preliminary Analyses

The mean, standard deviation, skewness, kurtosis, and Pearson correlation coefficients of study variables are reported in Table 2. All variables showed a univariate normal distribution, with values of skewness and kurtosis within the range of −1.0 and 1.0. Democratic parenting was positively and significantly correlated with positive well-being, vitality, and general health and negatively and significantly correlated with anxiety; environmental sensitivity was positively and significantly correlated with anxiety and negatively and significantly correlated with positive well-being, vitality, and general health; democratic parenting and environmental sensitivity were not significantly correlated with each other.

### 3.2. Relations Among Study Variables

All models had a good fit to the data: χ^2^(3) = 3.18, *p* = 0.36, CFI = 0.998, RMSEA = 0.014 (90% C.I., 0.000, 0.096). Parameter estimates are reported in Table 3.

#### 3.2.1. Positive Well-Being

Positive well-being was positively and significantly related to democratic parenting and negatively and significantly related to environmental sensitivity and gender. It was not significantly related to age and the interaction term, indicating that environmental sensitivity was not a significant moderator.

#### 3.2.2. Vitality

Vitality was positively and significantly related to democratic parenting and negatively and significantly related to environmental sensitivity, gender, and age. It was not significantly related to the interaction term, indicating that environmental sensitivity was not a significant moderator.

#### 3.2.3. Anxiety

Anxiety was negatively and significantly related to democratic parenting and positively and significantly related to environmental sensitivity and gender. It was not significantly related to age and the interaction term, indicating that environmental sensitivity was not a significant moderator.

#### 3.2.4. General Health

**Figure 2:**
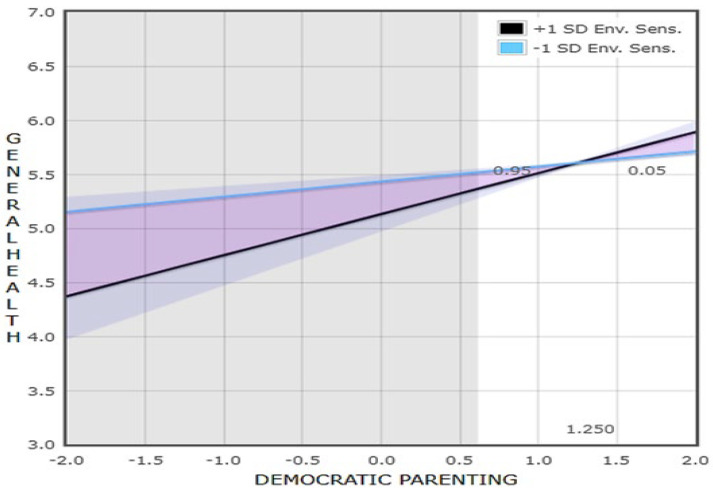
Simple slopes plot of the relation between Democratic Parenting and adolescents’ general health moderated by adolescents’ Environmental Sensitivity. The shaded area represents the region of significance in Democratic Parenting.

General health was positively and significantly related to democratic parenting and negatively and significantly related to environmental sensitivity. It was not significantly related to age and gender. The interaction term was significant, indicating that environmental sensitivity was a significant moderator. Simple slope plots for low and high levels of environmental sensitivity are reported in Figure 2. The results showed a positive and significant effect for low levels (B = 0.14, SE = 0.07, *p* = 0.046) and high levels (B = 0.38, SE = 0.07, *p* < 0.001) of environmental sensitivity. The slope patterns point to a “for worse” interaction where highly sensitive adolescents had lower general health when democratic parenting was low (see Figure 2). The RoS on the X test (±2 SD) showed a negative and significant effect for low levels of democratic parenting (B = −0.39, SE = 0.11, *p* = 0.001) but no significant effect for high levels of democratic parenting (B = 0.09, SE = 0.11, *p* < 0.408); thus, environmental sensitivity was only significantly associated with general health in the range of low to moderate levels of democratic parenting (shown by shaded area in Figure 2) pointing to a diathesis–stress (i.e., “for worse”) relationship. Additionally, the PoI index was equal to 0.05, suggesting further strong evidence for the diathesis–stress model. Both tests indicate that environmental sensitivity acts as a vulnerability factor in familiar environments characterized by low democratic parenting and does not significantly distinguish adolescents’ level of general health in familiar environments characterized by high democratic parenting.

## 4. Discussion

Parenting practices have been largely shown to contribute to adolescents’ adjustment and development [23,55]. However, the more recent literature has underlined that these associations might vary according to adolescents’ individual characteristics, such as their environmental sensitivity [8,11]. The general aim of this study was to analyze the specific role of democratic parenting and its interaction with environmental sensitivity on adolescents’ well-being (in terms of positivity, vitality, anxiety, and general health). Therefore, we tested three hypotheses within a sample of Italian adolescents. Specifically, we sought to elucidate whether the moderating role of environmental sensitivity in the association between democratic parenting practices and youths’ subjective well-being was consistent across the diathesis–stress, vantage sensitivity, and differential susceptibility models. Such analysis, in our view, might reveal whether democratic parenting practices are or are not universally beneficial for all youths, regardless of their personal susceptibility to contextual influences and proneness to respond to such external stimuli [38].

Results confirmed our first hypothesis (H1): Adolescents perceiving democratic practices from their parents exhibited low levels of anxiety and higher levels of positivity, vitality, and good general health. This is in line with studies testifying that authoritative parenting promotes children’s positive psychological assets, such as hope, self-efficacy, resilience, and optimism, and reduces youth anxiety [33,56]. Our data, thus, sustained the idea that the presence of a supportive and caring family is an important protective factor for adolescents’ well-being [57,58]. Particularly, it could be speculated that having parents who share democratic exchanges with their children encourages youth disclosure, creates occasions for constructive discussions, and contributes to establishing a positive familial climate, which promotes adolescents’ well-being and adjustment [6,59].

The findings also corroborated our second hypothesis (H2) since environmental sensitivity seemed to have a negative association with positive dimensions of well-being and a positive association with anxiety. This is in line with traditional research on environmental sensitivity reporting that high levels of this personal trait are associated with various maladaptive psychological outcomes, such as poor quality of life [60], anxiety, depression, poor social skills [45], higher levels of stress [61,62,63], and physical symptoms as well [62,64]. Therefore, our results are consistent with the literature that considers having high levels of sensitivity to the environment to be a risk factor for individual well-being. Of note, this study advanced extant knowledge by simultaneously considering diverse facets of well-being, including the understudied positivity and vitality.

Regarding the hypotheses about the moderating role of environmental sensitivity, results showed that it only conditioned the association between democratic parenting and youths’ general health. In detail, findings evidenced that adolescents reporting high levels of environmental sensitivity and perceiving low levels of democratic parenting tended to show lower general health than adolescents with low levels of environmental sensitivity. Overall, the current data seemed to support a diathesis–stress model (H3a) as more sensitive participants in this study had worse general health when they perceived that their parents were not or were little democratic. As previously suggested, consistent with this model, environmental sensitivity can be seen as a vulnerability factor leading to negative outcomes in the face of environmental adversities [36]. Specifically, based on our results, adolescents’ general health seems to be compromised when they are more sensitive to negative environmental stimuli, such as lower levels of democratic parenting.

Several shortcomings limit the interpretability of the present findings. First, the self-report nature of the measures may have increased the chance of inflated associations. For example, participants in the study might have over-reported their own perceptions of well-being [65]. Therefore, future research should include other approaches to measure well-being to mitigate this limitation. Second, the design of the study was cross-sectional, which might hinder the possibility of ascertaining temporal ordering and causality [66]. Thus, longitudinal designs are required in the future to better explain and catch developmental changes in the associations that were explored in this study. Additionally, several other factors may influence the associations among the study variables, such as schools or peer-related aspects. Future research should help identify other environmental influences that might have a role in these relations. Furthermore, although our data seem to corroborate a diathesis–stress perspective, these results may be the consequences of the specific variables considered. Therefore, the findings reported in our study only concern democratic parenting and well-being and should not be generalized to other parenting practices and/or outcomes. Lastly, it is worth noticing that the moderation effects were inconsistent across the other environmental sensitivity models (vantage sensitivity, differential susceptibility). This runs against previous studies showing that the three distinct theorized models (adverse sensitive, vantage sensitive, and differentially susceptible adolescents) may coexist in a given population [38]. In light of this, future works are needed to better explore children’s responsivity to democratic parenting practices and behaviors.

## 5. Conclusions

Despite these limitations, our results contributed to the literature in many ways. First, they confirmed the importance of a positive democratic family climate to both foster several dimensions of adolescents’ well-being (e.g., positivity, vitality, general health) and to discourage negative outcomes, such as anxiety. Second, our findings seem to indicate that being highly sensitive to the environment can be a potential risk factor for youth adjustment and call for greater attention to this individual characteristic during development. The most interesting contribution of our data is that they supported a diathesis–stress model of environmental sensitivity since participants in this study who were highly sensitive in the context of a low democratic family reported lower levels of general health.

In terms of implications, this study suggested that future intervention programs promoting youth well-being should adopt a socio-ecological perspective, in which both contextual (parenting practices) and individual aspects (environmental sensitivity) need to be considered and targeted. Based on our findings, specifically, it could be stated that parents should be prepared to be more aware of their children’s levels of environmental sensitivity and of the potential impact of their childrearing practices. More in detail, our results pointed out that mothers and fathers of highly sensitive children should be equipped to adopt attitudes and behaviors that make them feel valued and respected, as well as involved in familial processes and decisions. Practitioners implementing interventions to improve youths’ well-being, on the one hand, should help parents meet their children’s needs and respect their points of view and autonomy; on the other hand, they should consider adolescents’ level of environmental sensitivity as an important variable that might influence the potential outcomes or even the impact and effectiveness of their interventions. Practitioners, in addition, should support adolescents in developing adaptive strategies that may guide them while they are processing the experiences they live with their families.

## Figures and Tables

**Table 1:** Socio-demographic characteristics of participants (*n* = 321).

**Father’s Education**		**%**
	Primary School	5
	Middle School	40
	High School Diploma	44
	University Degree	9
	Post Lauream Degree	2
**Mother’s education**		**%**
	Primary School	1
	Middle School	32
	High School Diploma	52
	University Degree	12
	Post Lauream Degree	3
**Father’s occupation**		**%**
	Unemployed	6
	Self-employed worker	31
	Employee	58
	Retired	5
**Mother’s occupation**		**%**
	Unemployed	33
	Self-employed worker	25
	Employee	42
	Retired	<1
**Parents’ marital status**		**%**
	Partners	2
	Married	81
	Separated or divorced	9

**Table 2:** Mean, standard deviation, skewness, kurtosis, and Pearson correlation coefficients of study variables (*n* = 321).

	1	2	3	4	5	6	7
1 Democratic Parenting	–						
2 Environmental Sensitivity	−0.07	–					
3 Positive Well-Being	0.33 ***	−0.29 ***	–				
4 Vitality	0.36 ***	−0.27 ***	0.69 ***	–			
5 General Health	0.28 ***	−0.18 ***	0.47 ***	0.58 ***	–		
6 Anxiety	−0.34 ***	0.30 ***	−0.66 ***	−0.69 ***	−0.56 ***	–	
7 Age	0.03	−0.02	−0.05	−0.12 *	−0.02	0.10	–
8 Gender	−0.04	0.32 ***	−0.25 ***	−0.21 ***	−0.13 *	0.21 ***	0.04
*M*	18.37	63.68	13.64	14.13	13.14	17.17	16.83
*SD*	4.62	9.90	3.65	3.76	2.90	5.44	1.35
*S*	−0.74	−0.54	0.23	−0.33	−0.59	0.17	−0.22
*K*	−0.24	0.08	−0.18	−0.25	−0.03	−0.70	−0.76
*Observed Range*	6–24	30–82	4–24	4–22	4–18	6–30	14–19

Note. Gender was coded as 1 = male, 2 = female. * *p* < 0.05. *** *p* < 0.001.

**Table 3:** Parameter estimates of SEMs for well-being components (*n* = 321).

Model	*B*	*SE*	95% C.I.		*p*	*β*
			Lower Bound	Upper Bound		
*Positive Well-Being*						
Democratic Parenting	1.14	0.18	0.79	1.50	<0.001	0.31
Environmental Sensitivity	−0.79	0.19	−1.16	−0.41	<0.001	−0.22
DP × ES	0.12	0.18	−0.24	0.48	0.494	0.03
Age	−0.16	0.13	−0.42	0.10	0.239	−0.06
Gender	−1.47	0.44	−2.34	−0.60	0.001	−0.17
*R*^2^ = 0.21						
*Vitality*						
Democratic Parenting	1.27	0.18	0.91	1.64	<0.001	0.34
Environmental Sensitivity	−0.81	0.19	−1.19	−0.43	<0.001	−0.22
DP × ES	0.28	0.19	−0.09	0.64	0.140	0.07
Age	−0.35	0.14	−0.62	−0.08	0.010	−0.13
Gender	−1.16	0.45	−2.04	−0.27	0.010	−0.13
*R*^2^ = 0.22						
*General Health*						
Democratic parenting	0.76	0.15	0.47	1.06	<0.001	0.26
Environmental sensitivity	−0.43	0.16	−0.74	−0.11	0.008	−0.15
DP × ES	0.36	0.15	0.06	0.66	0.019	0.12
Age	−0.06	0.11	−0.28	0.16	0.570	−0.03
Gender	−0.59	0.37	−1.32	0.14	0.112	−0.09
*R*^2^ = 0.12						
*Anxiety*						
Democratic parenting	−1.76	0.27	−2.29	1.23	<0.001	−0.32
Environmental sensitivity	1.32	0.28	0.76	1.97	<0.001	0.24
DP × ES	−0.34	0.27	0.87	0.19	0.210	−0.06
Age	0.42	0.20	0.03	0.81	0.030	0.10
Gender	1.58	0.66	0.30	2.89	0.020	0.13
*R*^2^ = 0.22						

Note. DP × ES: interaction term “Democratic Parenting × Environmental Sensitivity”.

## Data Availability

All data sets are available upon request from the corresponding author.
